# Effective treatment in lung adenocarcinoma patient with brain metastases harboring novel *CLHC1/RNT4* intergenic region- *ALK* fusion: A case report

**DOI:** 10.1097/MD.0000000000029134

**Published:** 2022-04-08

**Authors:** Huanling Xia, Binbin Liang, Guoxiang Liu, Yingxue Qi, Ningning Luo, Mengmeng Li

**Affiliations:** a Department of Oncology, Jimo District People's Hospital, Qingdao, Shandong, China; b The Medical Department, Jiangsu Simcere Diagnostics Co., Ltd; Nanjing Simcere Medical Laboratory Science Co., Ltd; The State Key Lab of Translational Medicine and Innovative Drug Development, Jiangsu Simcere Diagnostics Co., Ltd, Nanjing, China.

**Keywords:** ALK TKI, brain metastases, lung adenocarcinoma, novel CLHC1/RNT4 intergenic region- ALK fusion

## Abstract

**Rationale:**

Anaplastic lymphoma kinase (ALK) fusion, an important oncogenic mutation, occurs in 3% to 7% of non-small cell lung cancer (NSCLC) cases, and EML4 is the most common partner gene. With the widespread application of next-generation sequencing (NGS), more gene breakpoint fusions have been discovered and functional fusion transcripts can provide targeted clinical benefits.

**Patient concerns and diagnosis:**

A 40-year-old woman was diagnosed with lung adenocarcinoma with brain metastases. A novel CLHC1/RNT4 intergenic region, ALK (Exon20-29) (abundance 39.97%), was identified using lung puncture tissue by NGS analysis (Simceredx), and results of immunohistochemistry and fluorescence in situ hybridization confirmed ALK fusion.

**Interventions and outcomes:**

The patient was administered oral crizotinib (250 mg bid) combined with endostar (30 mg d1-7) for 12 cycles from June 18, 2020. The patient's condition was controlled, and the curative effect was evaluated as stable disease (SD). Unfortunately, brain magnetic resonance images showed multiple nodules in the left cerebellar hemisphere, and chest computed tomography showed no significant changes in the progression of the disease. Subsequently, alectinib (600 mg bid) was administered on April 1, 2021. Brain lesions were significantly reduced and partial remission (PR) was achieved. No significant changes were observed in the lung lesions.

**Lessons:**

ALK fusion is a risk factor for brain metastasis (BM) in patients with advanced non-small NSCLC patients. In our case, a novel CLHC1/RNT4 intergenic region, ALK fusion, was identified for the first time in a lung adenocarcinoma patient with BM, who benefited from crizotinib and endostar sequential alectinib. Our case highlights the advantages of NGS for fusion detection and provides promising treatment options for NSCLC patients with BM harboring ALK fusions.

## 1 Introduction

Anaplastic lymphoma kinase (*ALK*) fusion, an important oncogenic mutation, occurs in 3% to 7% of non-small cell lung cancers (NSCLC), and *EML4* is the most common partner gene.^[1]^ With the widespread application of next-generation sequencing (NGS), more gene breakpoint fusions have been discovered, and functional fusion transcripts can bring targeted clinical benefits.^[2]^ Clinical trials have shown that patients with NSCLC with *ALK* fusion can obtain significant survival benefits through ALK tyrosine kinase inhibitor (ALK-TKI) treatment. To date, the FDA has approved 5 ALK-TKIs: crizotinib, ceritinib, alectinib, brigatinib, and lorlatinib.^[3,4]^ Herein, we first identified a novel *CLHC1/RNT4* intergenic region–*ALK* fusion in a patient with lung adenocarcinoma (LUAD) with brain metastasis (BM), who benefited from crizotinib and endostar sequential alectinib.

## 2 Case description

**Figure 1:**
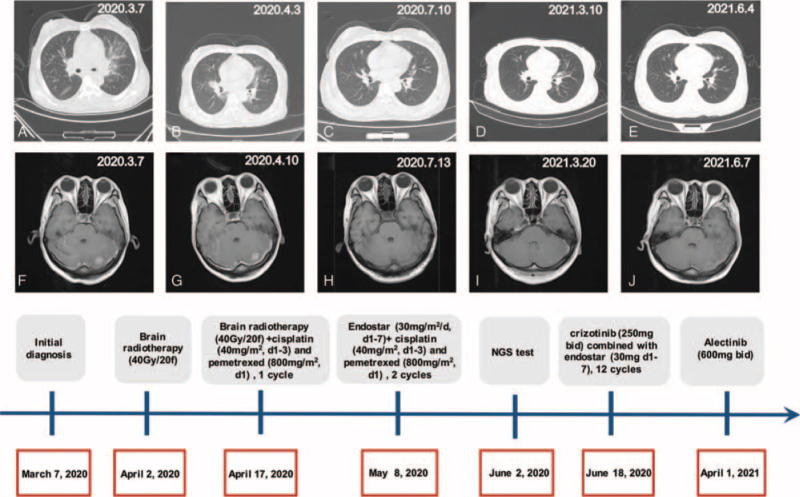
Chest computed tomography (CT) scan (A-E), magnetic resonance imaging (MRI) of the brain (F-J) and timeline of treatment. (A) CT of the lungs during the first physical examination. (B) Lung CT before receiving crizotinib treatment. (C) Lung CT after receiving crizotinib treatment. (D) Lung CT before receiving aletinib. (E) CT of the lungs after receiving aletinib. (F) Brain MRI of the first physical examination. (G) Brain MRI before crizotinib treatment (H) Brain MRI after crizotinib treatment. (I) Brain MRI before receiving aletinib. (J) Brain MRI after receiving aletinib.

A 40-year-old woman was admitted to the hospital on March 7, 2020, with “cough and hemoptysis”. Chest computed tomography (CT) revealed nodules in the upper lobe of the left lung with mediastinal lymph node metastasis (Fig. 1A). Brain magnetic resonance imaging (MRI) revealed BM (Fig. 1F). The neck lymph nodes were enlarged, and pathology of the neck lymph node biopsy showed LUAD. No driver gene mutations were found by ARM-PCR in the patient's blood in the hospital. Brain radiotherapy (40 Gy/20 f) was performed from April 2, 2020, to May 6, 2020. During brain radiotherapy, cisplatin (40 mg/m^2^, D1-3) and pemetrexed (800 mg/m^2^/d) were administered for 1 cycle of chemotherapy from April 17, 2020. Unfortunately, her condition worsened, her breathing was difficult, and chest and back pain were present. Positron emission tomography-CT revealed a significant increase in pericardial effusion. The patient received 2 cycles of endostar (30 mg/m^2^/d, d1-7) combined with cisplatin (40 mg/m^2^, D1-3) and pemetrexed (800 mg/m^2^/d) on May 8, 2020.

**Figure 2:**
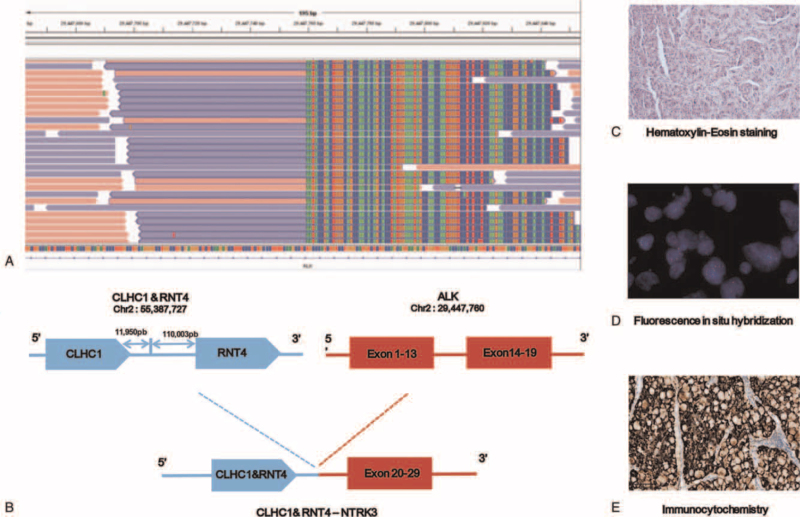
Next-generation sequencing findings of CLHC1/RNT4 intergenic region- ALK fusion. (A) The Integrative Genomics Viewer snapshot of CLHC1/RNT4 intergenic region- ALK. (B) Schematic representation of the CLHC1/RNT4 intergenic region- ALK fusion protein domain structure. Histopathologic stains from the pulmonary biopsy (C-E). (C) Hematoxylin-Eosin staining. (D) Fluorescence in situ hybridization. (E) Immunocytochemistry staining.

A novel *CLHC1/RNT4* intergenic region, *ALK* (Exon20-29) (abundance 39.97%), was identified using lung puncture tissue by NGS analysis (Simceredx) in June 2020, and the results of immunohistochemistry and fluorescence in situ hybridization confirmed *ALK* fusion (Fig. 2). The patient then started oral crizotinib (250 mg bid) combined with endostar (30 mg d1-7) for 12 cycles from June 18, 2020. The patient's condition was controlled and the curative effect was evaluated as stable disease (SD) (Fig. 1C and H). Unfortunately, the patient felt dizzy and unwell in March 2021. Brain MRI showed multiple nodules in the left cerebellar hemisphere (Fig. 1I), and chest CT showed no significant changes (Fig. 1D), revealing the progression of the disease. Alectinib (600 mg twice daily) was administered on April 1, 2021. Brain lesions were significantly reduced, and partial remission (PR) was achieved (Fig. 1J). No significant changes were observed in the lung lesions had no significant change) (Fig. 1E). The timeline of treatment and changes in the CT scan and MRI are shown in Figure 1.

## 3 Discussion

In our case, we identified a novel *CLHC1/RNT4* intergenic region-*ALK* fusion in a LUAD patient with BM, which retained the entire kinase domain (exon 20-29) of *ALK* (Fig. 2B). Because functional intergenic fusions can bring corresponding clinical benefits, they are receiving increasing attention. Compared with the limited traditional detection methods, the application of NGS has accelerated the discovery of novel *ALK* fusions, and the requirements for samples have gradually broadened.^[5]^

The novel *ALK* fusion gene was confirmed as a functional fusion transcript by fluorescence in situ hybridization and immunohistochemistry (Fig. 1L). Lung cancer patients with intergenic *ALK* fusions respond to ALK-TKIs.^[6–8]^*ALK* fusion is a risk factor for brain patients with advanced NSCLC.^[9]^ Clinical trials of TKI in NSCLC patients with BM have shown a high percentage of objective responses, prolonged PFS, and improved quality of life. Studies have reported that 6 patients with NSCLC and brain metastases received 100% intracranial ORR (4 cases of PR and 2 cases of CR) after receiving osimertinib and bevacizumab combination treatment.^[10]^ The patient in our case with intergenic *ALK* fusion had a good response to ALK TKI plus endostar.

## 4 Conclusion

In conclusion, a novel *CLHC1/RNT4* intergenic region, *ALK* fusion, was identified for the first time in a LUAD patient with BM, who benefited from crizotinib and endostar sequential alectinib. Our case highlights the advantages of NGS for fusion detection and provides promising treatment options for NSCLC patients with BM harboring *ALK* fusions.

## Acknowledgments

The authors thank Mr. Chuang Qi, Ms. Tingting Sun, Ms. Xueyu Yang, Mr. Wanglong Deng, Mr. Guanghua Lu, and Mr. Ran Ding from Simceredx for their assistance.

## Author contributions

All authors made substantial contributions to the conception of this study. All authors approved the final manuscript as submitted and agreed to be accountable for all aspects of the work.

**Conceptualization:** Huanling Xia.

**Data curation:** Binbin Liang.

**Writing – original draft:** Yingxue Qi, Guoxiang Liu.

**Writing – review & editing:** Ningning Luo, Mengmeng Li.

## References

[1] Solomon B Varella-Garcia M Camidge DR. ALK gene rearrangements: a new therapeutic target in a molecularly defined subset of non-small cell lung cancer. J Thorac Oncol 2009;4:1450–4. 20009909 10.1097/JTO.0b013e3181c4dedb

[2] Li W Liu Y Li W. Intergenic breakpoints identified by DNA sequencing confound targetable kinase fusion detection in NSCLC. J Thorac Oncol 2020;15:1223–31. 32151779 10.1016/j.jtho.2020.02.023

[3] Vavalà T Novello S. Alectinib in the treatment of ALK-positiven on-small cell lung cancer: an update on its properties, efficacy, safety and place in therapy. Ther Adv Med Oncol 2018;10:1758835918789364. 30090122 10.1177/1758835918789364 PMC6077883

[4] Paliouras AR Buzzetti M Shi L. Vulnerability of drug-resistant EML4-ALK rearranged lung cancer to transcriptional inhibition. EMBO Mol Med 2020;12:111. 10.15252/emmm.201911099 PMC7338803 32558295

[5] Solomon JP Benayed R Hechtman JF. Identifying patients with NTRK fusion cancer. Ann Oncol 2019;30: (Suppl 8): viii16–22. 10.1093/annonc/mdz384 32223934

[6] Chen X Zhao G Zhong P Zhang M Chen R Zhang D. Chr2 30297612-ALK, a novel intergenic fusion with exon18 of ALK, responds to crizotinib. Clin Lung Cancer 2020;21:e564–6. 32576444 10.1016/j.cllc.2020.04.014

[7] Zhang J Zou C Zhou C. A novel Linc00308/D21S2088E intergenic region ALK fusion and its enduring clinical responses to crizotinib. J Thorac Oncol 2020;15:1073–7. 32217130 10.1016/j.jtho.2020.03.009

[8] Fei X Zhu L Zhou H Qi C Wang C. A novel intergenic region between CENPA and DPYSL5-ALK Exon 20 fusion variant responding to crizotinib treatment in a patient with lung adenocarcinoma. J Thorac Oncol 2019;14:e191–3. 31445731 10.1016/j.jtho.2019.04.012

[9] Wang H Wang Z Zhang G. Driver genes as predictive indicators of brain metastasis in patients with advanced NSCLC: EGFR, ALK, and RET gene mutations. Cancer Med 2020;9:487–95. 31769228 10.1002/cam4.2706 PMC6970058

[10] Yu HA Schoenfeld AJ Makhnin A. Effect of osimertinib and bevacizumab on progression-free survival for patients with metastatic EGFR-mutant lung cancers: a phase 1/2 single-group open-label trial. JAMA Oncol 2020;6:1048–54. 32463456 10.1001/jamaoncol.2020.1260 PMC7256866

